# Integrating Domain Specific Knowledge and Network Analysis to Predict Drug Sensitivity of Cancer Cell Lines

**DOI:** 10.1371/journal.pone.0162173

**Published:** 2016-09-08

**Authors:** Sebo Kim, Varsha Sundaresan, Lei Zhou, Tamer Kahveci

**Affiliations:** 1 Department of Computer and Information Science and Engineering, University of Florida, Gainesville, FL, United States of America; 2 Department of Molecular Genetics and Microbiology College of Medicine, University of Florida, Gainesville, FL, United States of America; Texas A&M University College Station, UNITED STATES

## Abstract

One of fundamental challenges in cancer studies is that varying molecular characteristics of different tumor types may lead to resistance to certain drugs. As a result, the same drug can lead to significantly different results in different types of cancer thus emphasizing the need for individualized medicine. Individual prediction of drug response has great potential to aid in improving the clinical outcome and reduce the financial costs associated with prescribing chemotherapy drugs to which the patient’s tumor might be resistant. In this paper we develop a network based classifier (NBC) method for predicting sensitivity of cell lines to anticancer drugs from transcriptome data. In the literature, this strategy has been used for predicting cancer types. Here, we extend it to estimate sensitivity of cells from different tumor types to various anticancer drugs. Furthermore, we incorporate domain specific knowledge such as the use of apoptotic gene list and clinical dose information in our method to impart biological significance to the prediction. Our experimental results suggest that our network based classifier (NBC) method outperforms existing classifiers in estimating sensitivity of cell lines for different drugs.

## Introduction

American Cancer Society estimates that in 2015, there will be 1,658,370 new cancer cases diagnosed and 589,430 cancer deaths in the US. In over four decades of research, cancer therapy has evolved from surgery, radiotherapy, chemotherapy, endocrine therapy to targeted and combined therapy. Surgery is known to be most effective in complete excision of solid tumors but there are concerns of morbidity and mortality associated with it. Combinatorial approaches of radiation and chemotherapy have reduced the use of invasive surgical excision of tumors [1]. A majority of conventional chemotherapeutic drugs target rapidly dividing cells. However, these drugs often do not specifically target the cancer site causing systemic off target effects. Based on increasing knowledge of specific tumor subtypes and their molecular basis, new targets including growth factors, signaling molecules, cell-cycle proteins, immunotherapeutic agents, modulators of apoptosis and molecules that promote angiogenesis or target hypoxia have shown promise in cancer therapy [1].

One of fundamental challenges in cancer studies is that varying molecular characteristics of different tumor types may lead to resistance to certain drugs. As a result, the same drug can lead to significantly different results in different types of cancer thus emphasizing the need for individualized medicine. Individual prediction of drug response has great potential to aid in improving the clinical outcome and reduce the financial costs associated with prescribing chemotherapy drugs to which the patient’s tumor might be resistant. Techniques to improve the clinical outcome of chemotherapeutic drugs include improved genetic profiling and tumor characterization. Developing targeted drugs to specific kinases for example or targeting mutations observed in specific tumor types has also shown decrease in drug resistance and off target effects [2]. Discovery of predictive biomarkers have already helped in understanding the response of the tumors to drugs that work on specific molecular targets. However, this information is still missing for many of the tumor types. In vitro cell culture models have been used widely by researchers to understand in vivo responses to drugs since decades. cDNA microarray studies and gene expression levels have been used as tools to predict chemosensitivity of cancer cell lines and primary tumor cells as well [3]. As new cancer types and cell lines are being identified everyday, screening of each of the cancer cell lines against the available drugs requires extensive manual labor and can be time consuming.

A promising approach to overcome the limitations with in vitro drug sensitivity prediction is to employ computational techniques. Qin et al, for instance, have developed a network flow-based method to predict anticancer drug sensitivity using the drug response data from the Cancer Genome Project (CGP) [4]. It exploits the topological structure of pathways whose activity difference before and after drug treatment is used as a measure of drug response. Park et al proposed a novel outlier-resistant method for identifying sensitivity-specific biomarkers for individual patients and predicting anticancer drug sensitivity [5]. In reality, genomic datasets usually include outliers which may notably affect the result of analysis. In this method, robust Mahalanobis distance in robust principal component space controls outliers of gene expression levels and drug response in high dimensional space. Costello et al compared various drug sensitivity prediction algorithms and discussed the advantages of Bayesian multitask multiple kernel learning (MKL) methodology over others [3]. MKL method exploits four machine learning principles: kernelized regression, multiview learning, multitask learning, and Bayesian inference. Kernelized regression can capture non-linear relationships between genomic features, and drug sensitivities of cell line. Multiview learning principle integrates heterogeneous input data into a single model. Multitask learning is the sharing of information between drugs, which implies simultaneous modeling of drug sensitivities across all the drugs. Additionally, Bayesian inference learned all model parameters to handle the uncertainty from the small sample size. Berlow et al proposed a novel sensitivity prediction approach based on functional perturbation data that incorporates the drug protein interaction information and sensitivities to a training set of drugs with known targets [6]. According to Berlow, “the proposed framework provides a unique input-output based methodology to model a cancer pathway and predict the effectiveness of targeted anti-cancer drugs”. Zhang et al proposed a dual-layer integrated cell line-drug network model. It uses both cell line similarity network (CSN) data and drug similarity network (DSN) data to predict the drug response of a given cell line [7]. Zhang noted that “proposed dual-layer integrated cell line-drug network model combines the predictions from the individual CSN and DSN layers, and predicts a response of a cell line to a drug based on how similar cell lines (CSN) respond to similar drugs (DSN)”. Their method is not significantly affected by the huge dimensionality of gene expression features because the model only requires correlations between cell lines or drugs as input.

In this paper we develop a network based classifier (NBC) method for predicting sensitivity of cell lines to anticancer drugs from transcriptome data. In the literature, this strategy has been used for predicting cancer types. Here, we extend it to estimate sensitivity of cells from different tumor types to various anticancer drugs. Furthermore, we incorporate domain specific knowledge such as the use of apoptotic gene list and clinical dose information in our method to impart biological significance to the prediction. Our experimental results suggest that our NBC method outperforms existing classifiers in estimating sensitivity of cell lines for different drugs. We also show that network models created by NBC method is able to guide the selection of genes that are worth further investigation for certain anticancer drugs.

## Results

In this section we evaluate the performance of our method for predicting drug sensitivity. Below, we first describe our experimental set up. We then present out results.

**Implementation details** We implemented NBC in the C programming language, and used “scikit-learn” library [8] for the other predictors and *χ*^2^ feature selection [9]. Mehmet Gonen implemented Bayesian multitask multiple kernel learning (BMTMKL) in R language [3].

The transcriptome in the CCLE dataset contains *m* = 18,926 genes in total, and the transcriptome in the GDSC dataset contains *m* = 19,930 genes in total. In our experiments, we selected a subset of *m*′ = 100 genes from these genes using the *χ*^2^ feature selection method unless otherwise stated. When we chose genes less than 100, the prediction performance of the model got worse. When we chose genes more than 100, the prediction performance remained the same or got worse. That is why we selected 100 genes.

We used 5-fold cross-validation (CV) to assess the performance of our method and the competing predictors and also to tune the parameters of predictors. 5-fold cross-validation (CV) randomly partitions the original dataset into five subsets. We use four of these subsets for training, and the remaining one to test the accuracy of the predictor. We repeat the cross-validation (CV) process 5 times, each with a different test dataset as the validation data. We then report the average of the resulting five accuracies. We repeat the CV this way 100 times and report the average balanced accuracy (BAC).

### Comparison of NBC using Support Vector regression (SVR) to existing predictors

Our first experiment compares the accuracy of the NBC method using SVR (non-linear) predictor with a broad spectrum of existing state-of-the-art methods. More specifically, we compare it with eight different methods; NBC using Ridge regression (linear), Support Vector Machine (SVM) using linear and RBF (non-linear) predictors, Random Forest (RF), Gaussian Naive Bayes (GNB), *k*-nearest neighbor (kNN) methods, ElasticNet, and Bayesian multitask multiple kernel learning (BMTMKL). NBC is a network-based method, while the classical learning algorithms are single-gene-based. Network-based method exploits the interaction among genes through their expression levels. Thus, we expect the network-based method to yield higher accuracy than the classical learning algorithms.

For each drug, we perform double nested 5-fold cross-validation and compute the average balanced accuracy (BAC) and Matthews correlation coefficient (MCC). We repeat this 100 times each time with a different partitioning of the training and test samples and report the average result. In the double cross validation, the inner 5-fold CV tunes the parameters of predictors. The outer 5-fold CV measure the BAC or MCC of the resulting predictor. To avoid overfitting problem, we split the data randomly into two subsets. We use the first one to train the model and the second one to test the model. We call the first one as training data, the second one as test data. We do not know the appropriate parameters to train the model. Thus, we again split the training data randomly into two subsets. We use the first one to train the parameters in the inner CV, the second one to validate the parameters in the inner CV.

Notice that each learning algorithm has a unique set of parameters. NBC has one parameter that is a correlation threshold used to construct network graph models. Linear Support Vector Machine (SVM) has a penalty parameter *C* to control the trade-off between the error obtained on the training data and margin maximization. Support Vector Machine (SVM) using radial basis function (RBF) kernel has a penalty parameter *C* and also a parameter *γ* to govern how far the influence of a single training point reaches. Random Forest (RF) has many parameters. Among them, we only learn the parameter describing the number of decision trees. Gaussian Naive Bayes (GNB) is the only predictor that does not have any parameter. *K*-nearest neighbor (kNN) has a parameter *k* that defines the number of neighbors used in classifying a test sample. ElasticNet has a tuning parameter *α*, which controls the strength of the penalty term. It controls the trade-off between fitting a linear model, and shrinking the coefficients. ElasticNet also has a parameter *l*1_*ratio* to control the ratio of L1 penalty used. If it is zero, then the penalty is an L2 penalty. If it is one, then the penalty is an L1 penalty. If it is between zero and one, then the penalty is a combination of L1 and L2. Bayesian multitask multiple kernel learning (BMTMKL) has two parameters *α* and *β* for gamma prior to control the trade-off between the goodness for obtaining sparsity and the goodness for small sample size problems. We use the parameter spaces for each of the predictors as shown in the Table 1.

**Table 1:** Parameter spaces for each of the predictors.

Method	Parameter	Values
NBC	correlation threshold	{0.4, 0.46, …, 0.88, 0.94}
Linear SVM	*C*	{10^−5^, 10^−4^, …, 10^3^, 10^4^}
SVM-RBF	*C*	{10^−5^, 10^−4^, …, 10^3^, 10^4^}
SVM-RBF	*γ*	{10^−5^, 10^−4^, …, 10^3^, 10^4^}
RF	*n*	{2, 4, …, 18, 20 }
kNN	*k*	{1, 3, …, 17, 19 }
ElasticNet	*α*	{10^−2^, 10^−1^, …, 10^1^}
ElasticNet	*l*1_*ratio*	{0.0, 0.25, …, 0.75}
BMTMKL	*α*	{10^−10^, 10^−5^, …, 10^10^}
BMTMKL	*β*	{10^−10^, 10^−5^, …, 10^10^}

**Fig 1:**
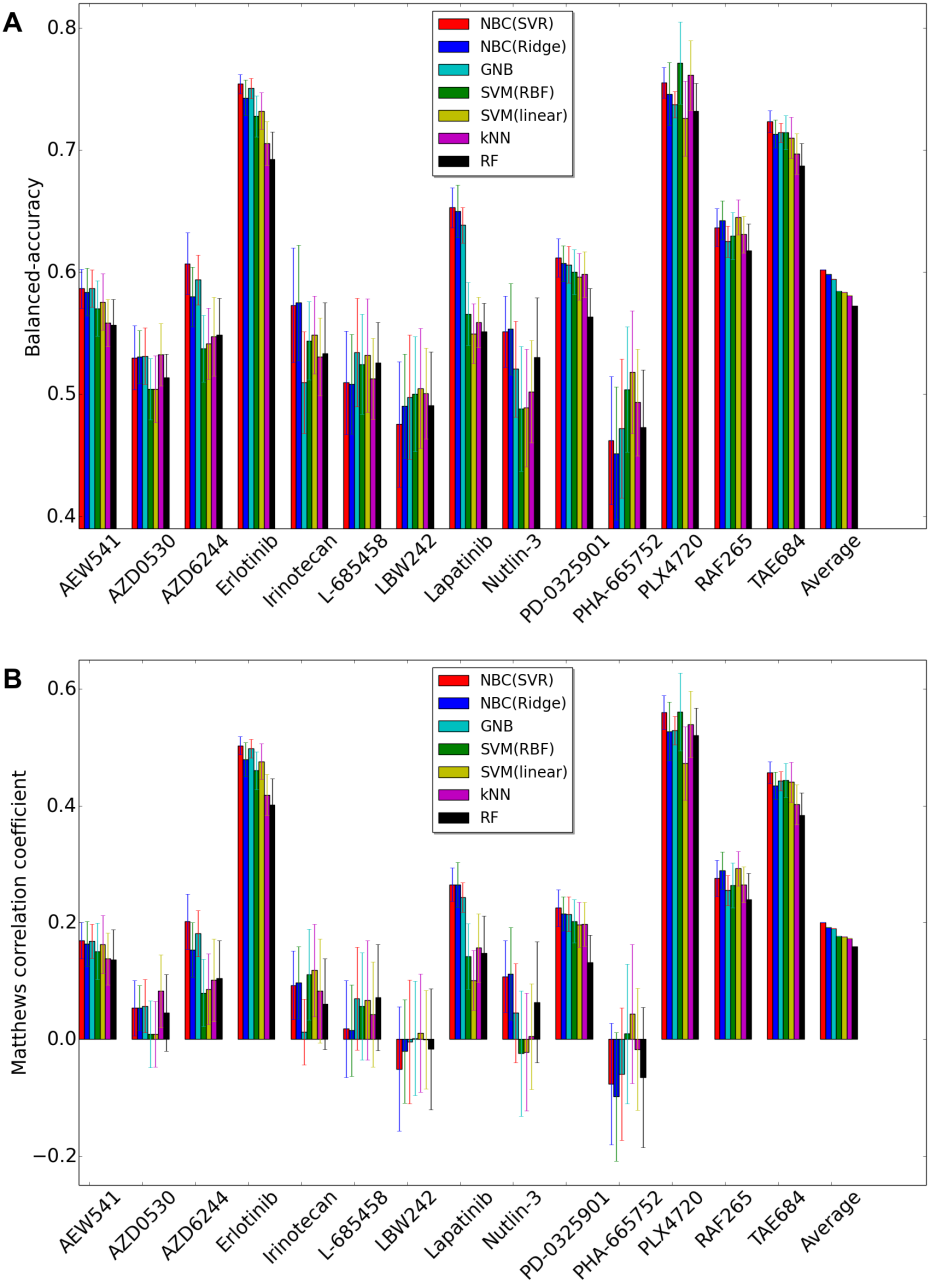
Performance comparison using CCLE dataset between NBC using SVR, NBC using Ridge regression, Gaussian Naive Bayes, SVM-RBF, SVM-Linear, K-Nearest-Neighbor, Random Forest. X-axis represents drug names and average of all drugs, and y-axis represents BAC or MCC. (A)Balanced Accuracy (BAC). (B)Matthews correlation coefficient (MCC).

Fig 1 shows the performance comparison when we use CCLE dataset. The results of BAC (Fig 1A) demonstrate that the accuracy of NBC using Support Vector regression (SVR) is the highest among all competing methods considering the average over all 14 drugs. The second best is NBC using Ridge regression (linear) predictor, and Gaussian Naive Bayes (GNB) has the highest accuracy among single-gene-based methods. In 6 out of the 14 drugs, NBC using Support Vector regression (SVR) is the winner among the six different methods we tested. Linear SVM yields the best accuracy in three drugs, NBC using Ridge regression (linear) predictor is the best in two different drugs, GNB is the best in one drug, SVM with radial basis function (RBF) kernel is the best in one drug, and kNN is the best in one drug. More complex models (i.e., low-bias models) show the performance at least comparable to the performance of less complex models for all drugs except for PHA-665752. Linear SVM is a certain winner for the drug PHA-665752. It means that unnecessary complexity can drop the prediction performance.

For the standard deviation of the accuracy over 100 trials, NBC using SVR regularly shows stable performance. NBC using SVR is the best in ‘Erlotinib’, ‘Lapatinib’, and ‘TAE684’ in terms of both the balanced accuracy (BAC) and the standard deviation of the performance.

**Fig 2:**
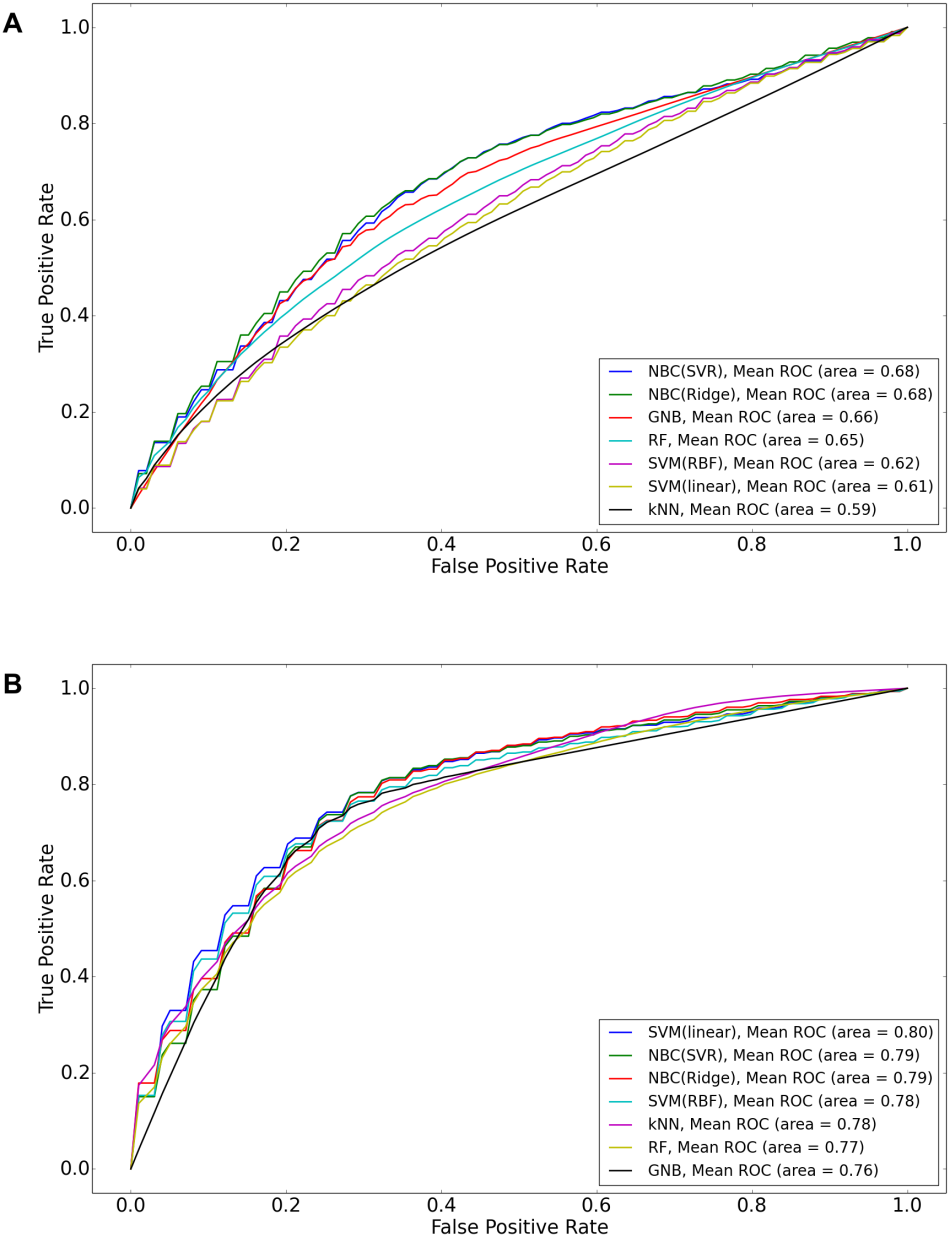
Receiver operating characteristic (ROC) curves using CCLE data set. X-axis represents false positive rate, and y-axis represents true positive rate. (A)ROC curve for drug Lapatinib. (B)ROC curve for drug Erlotinib.

The results of Matthews correlation coefficient (MCC) (Fig 1B) is almost similar with the results of BAC (Fig 1A). Only small difference between two figures is MCCs of SVM(RBF), SVM(linear) are greater than MCCs of NBC(SVR), NBC(Ridge) for Irinotecan, and MCC of NBC(SVR) is similar to that of SVM(RBF) for PLX4720. Fig 2 shows two Receiver operating characteristic (ROC) curves for Erlotinib, and Lapatinib. For Erlotinib, even if BAC of SVM(linear) was less than that of NBC(SVR), SVM(linear) in the ROC figure has the highest Area Under Curve (AUC). For Lapatinib, NBC(SVR) has the highest AUC. Balanced accuracy or MCC is based on one specific cutpoint, while ROC tries all of the cutpoint and plots the sensitivity and specificity. So when we compare the balanced accuracy or MCC, we are comparing based on some cutpoint. The balanced accuracy or MCC varies from different cutpoint. SVM (linear) has higher AUC in Fig 2B though balanced accuracy/MCC of SVM (linear) for Erlotinib is lower than that of NBC (SVR) in Fig 1. That shows NBC (SVR) is better in a particular cutpoint in measuring balanced accuracy or MCC metric. However, SVM (linear) is better when we have to take account of not a particular cutpoint but all cutpoints overall.

**Fig 3:**
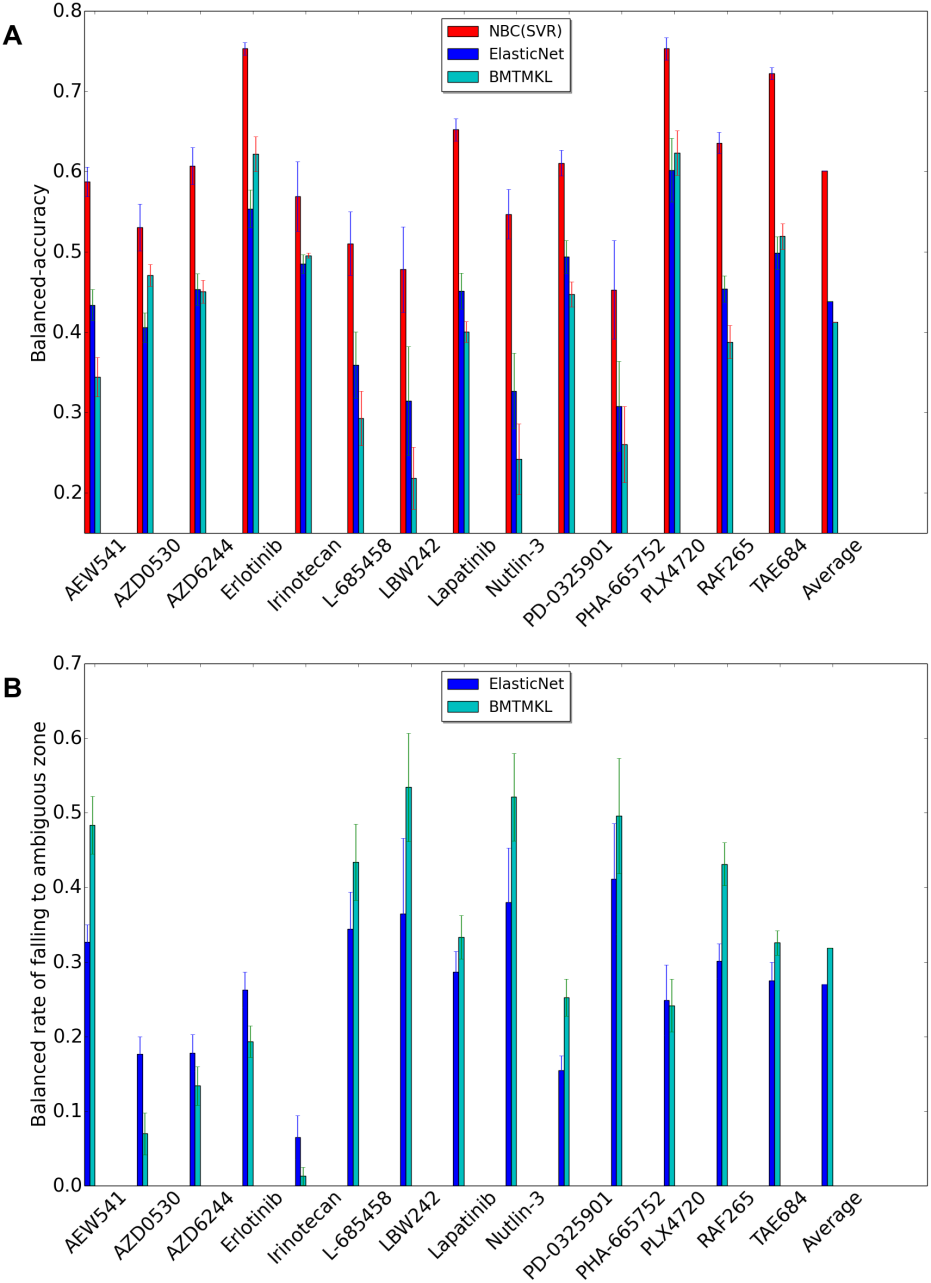
Performance comparison using CCLE dataset between NBC using SVR, ElasticNet, BMTMKL. X-axis represents drug names and average of all drugs, and y-axis represents BAC or balance rate of falling to ambiguous zone. (A)Balanced Accuracy (BAC). (B)Balanced rate of falling to ambiguous zone.

Fig 3 shows the performance comparison between NBC(SVR), ElasticNet, and Bayesian multitask multiple kernel learning (BMTMKL) when we use CCLE dataset. Recall that we have two clinical cutoff values for each drug. If the −log(*EC*50) is above the sensitive cutoff, we call this cell line to be *sensitive* to that drug. Likewise, if the −log(*EC*50) is below the resistant cutoff, we say that this cell line is *resistant* to that drug. If the −log(*EC*50) is between resistant and sensitive cutoffs, that cell line does not clearly belong to any of the two classes. We ignore such cell lines in our experiments. Therefore, all samples are either resistant or sensitive. However, regression methods yield the ambiguous class when predicted value lies between two clinical cutoff values. Thus, the shape of confusion matrix for regression methods in our experiments is two (actual sensitive, and actual resistant) by three (predicted sensitive, predicted ambiguous, and predicted resistant). We modify the formula of balanced accuracy for regression methods. We call when the sample is actual sensitive and predicted sensitive as true sensitive (*TS*), when the sample is actual sensitive and predicted ambiguous as false ambiguous from sensitive (*FAS*), when the sample is actual sensitive and predicted resistant as false resistant (*FR*), when the sample is actual resistant and predicted sensitive as false sensitive (*FS*), when the sample is actual resistant and predicted ambiguous as false ambiguous from resistant (*FAR*), and when the sample is actual resistant and predicted resistant as true resistant (*TR*).

$$ModifiedBAC=\frac{0.5*TS}{TS+FAS+FR}+\frac{0.5*TR}{FS+FAR+TR}$$

We also define balanced rate of falling to ambiguous zone as $\frac{0.5*FAS}{TS+FAS+FR}+\frac{0.5*FAR}{FS+FAR+TR}$. The balanced rate of falling to ambiguous zone of classification methods must be zero because classification methods do not predict the sample as ambiguous. NBC (SVR) method classifies each samples to either resistant or ambiguous class. Thus, unlike ElasticNet and BMTMKL, it guarantees to never produce an ambiguous classification. It always has a rate of zero for falling into ambiguous class.

Fig 3A presents BACs, and Fig 3B presents the balanced rate of falling to ambiguous zone. A natural outcome is the higher the balanced rate of falling to ambiguous zone is, the less the BAC is. The BAC of regression methods is inherently inferior compared to the BAC of classification methods because a lot of samples that belong to predicted ambiguous class hurts the performance.

**Fig 4:**
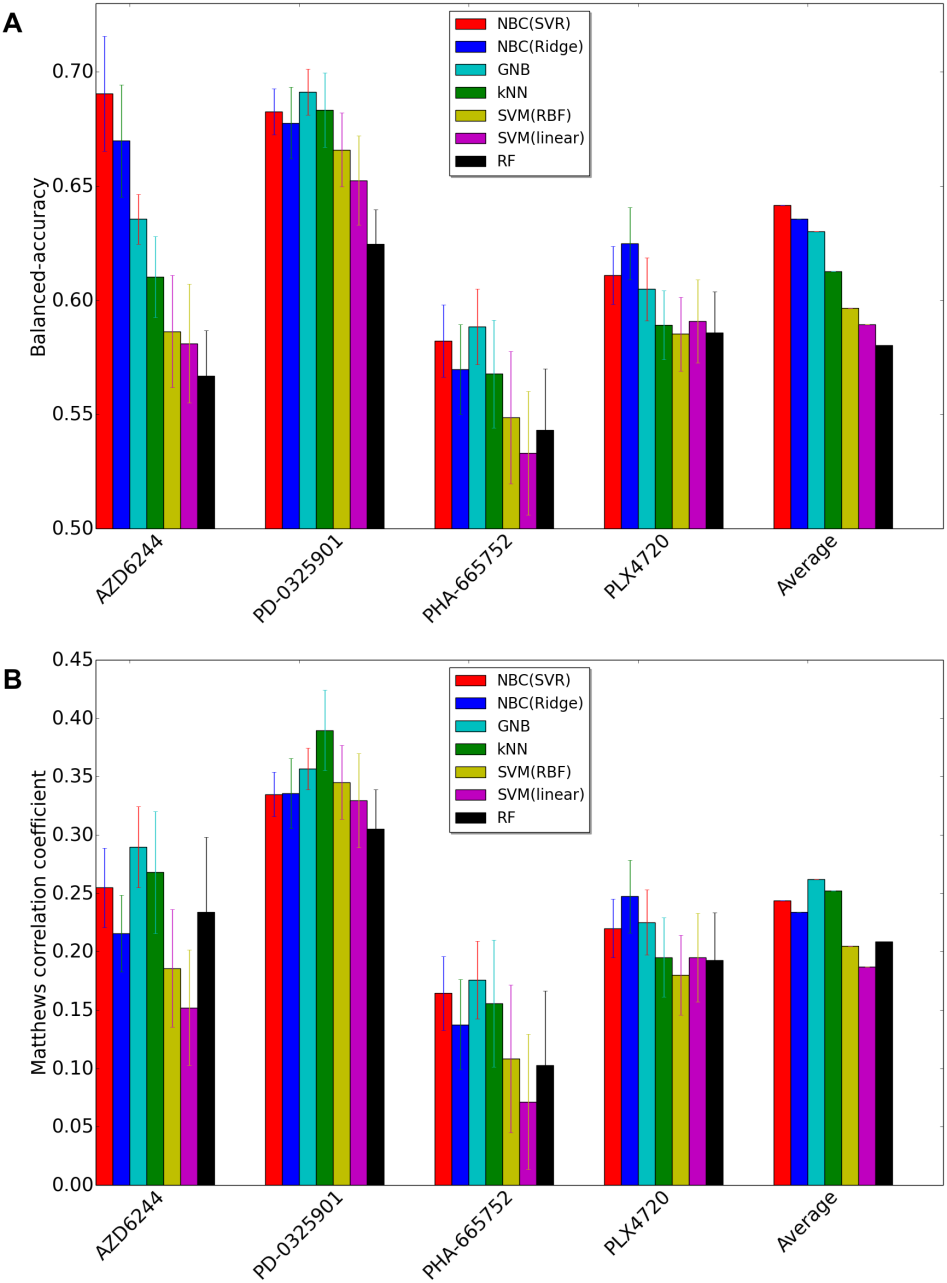
Performance comparison using GDSC dataset between NBC using SVR, NBC using Ridge regression, Gaussian Naive Bayes, SVM-RBF, SVM-Linear, K-Nearest-Neighbor, Random Forest. X-axis represents drug names and average of all drugs, and y-axis represents BAC or MCC. (A)Balanced Accuracy (BAC). (B)Matthews correlation coefficient (MCC).

Fig 4 shows the performance comparison when we use GDSC dataset. The results of BAC (Fig 4A) demonstrates that the accuracy of NBC using Support Vector regression (SVR) is the highest among all competing methods considering the average over all four drugs. However, the MCCs of NBC(SVR) for AZD6244 and PD-0325901 are less than those of GNB, kNN. Due to the big loss in AZD6244 and PD-0325901, NBC(SVR) yields the top rank to GNB in Fig 4B. It is true that often when the balanced accuracy of a confusion matrix is higher than another confusion matrix, MCC of the confusion matrix is also higher than that of another confusion matrix. However, this does not have to hold all the time. The disagreement between the two measures can happen when the two classes have very different sizes. For instance, a few misclassifications in the small class can decrease the balanced accuracy rapidly, while decreasing MCC gradually. NBC (SVR) tries to minimize the misclassification mistake on the small class, which makes the balanced accuracy high and hurts the MCC, while GNB tends to allow the misclassification mistake on the small class assuring a better classification performance on the big class. In conclusion, if we particularly care about correct classification of the smaller sized class, NBC outperforms GNB, otherwise it is the opposite.

**Fig 5:**
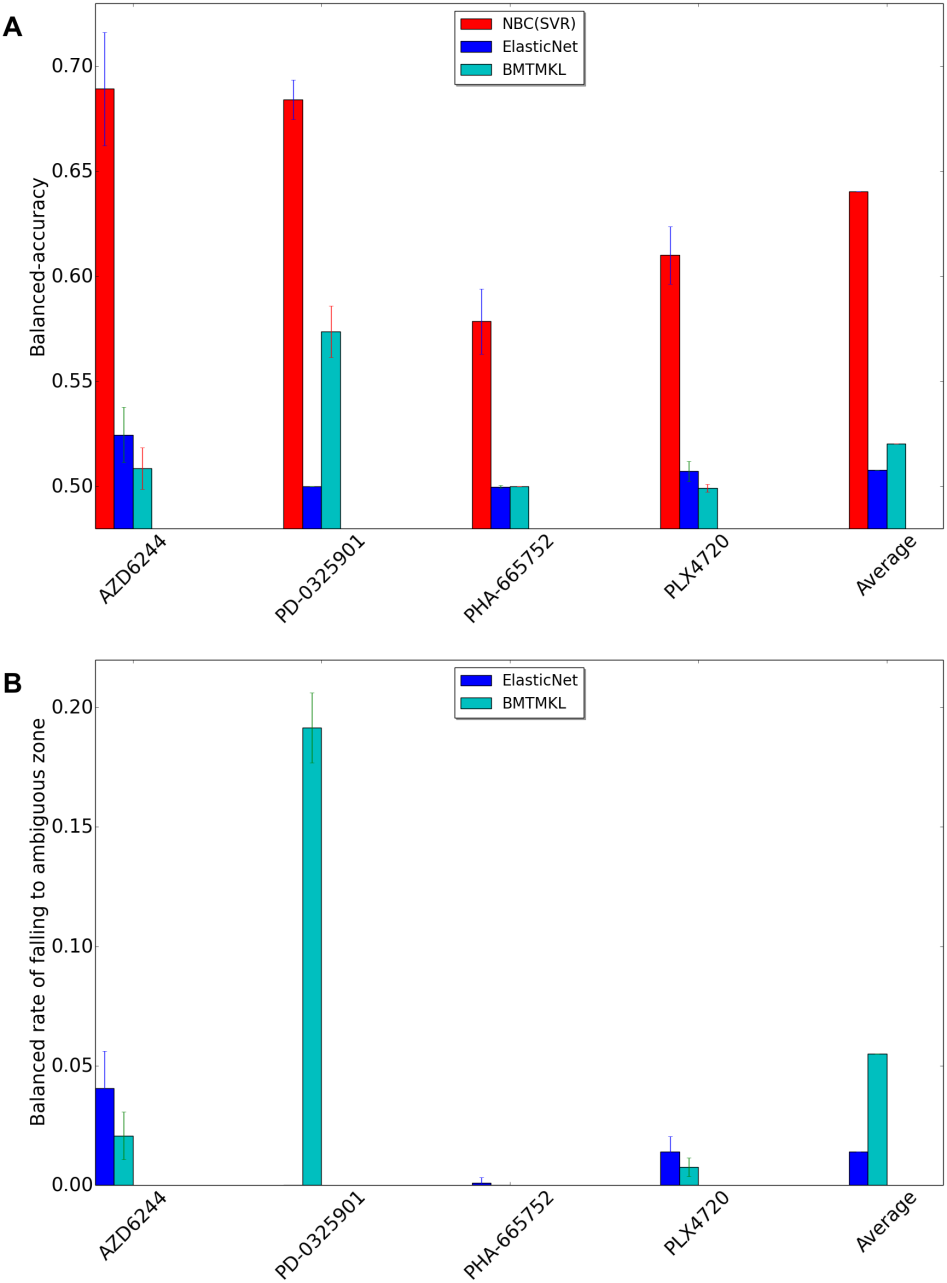
Performance comparison using GDSC dataset between NBC using SVR, ElasticNet, BMTMKL. X-axis represents drug names and average of all drugs, and y-axis represents BAC or balance rate of falling to ambiguous zone. (A)Balanced Accuracy (BAC). (B)Balanced rate of falling to ambiguous zone.

Fig 5 shows the performance comparison between NBC(SVR), ElasticNet, and Bayesian multitask multiple kernel learning (BMTMKL) when we use GDSC dataset. Like the preceding result using CCLE, the BAC of regression methods is inferior compared to the BAC of classification methods.

Thus we conclude that NBC using SVR (non-linear) predictors yields the most accurate predictions about drug sensitivity. In the rest of our experiments, we focus on this method.

### Detailed evaluation of the NBC method

In our previous experiment, we observed that the NBC method using SVR (non-linear) predictors yields the highest accuracy among all competing methods tested. For simplicity, we will use the name NBC to indicate the NBC method with SVR (non-linear) predictors in the rest of the experiments. Note that, the NBC method relies on a parameter, correlation threshold for building the network model (see Materials and Methods section). This parameter governs the density of the resulting network model. Thus, it has the potential to affect the prediction accuracy of NBC. In this experiment, we focus on the NBC method to understand how this parameter affects the accuracy of NBC for different drugs.

More specifically, for each of the 14 different drugs, we evaluate the accuracy of NBC by varying the correlation thresholds (from 0.40 to 0.94) at 10 equally spaced correlation values. We perform 5-fold CV where we use 80% of the samples for training and the remaining 20% for testing at each iteration of the CV. Unlike the previous experiment, here we do not perform double nested CV as we do not need to tune the correlation threshold parameter. For each value of the correlation threshold and drug combination, we repeat this experiment 100 times, each time with a different partitioning of the training and test samples. We report the average BAC value over all the 100 experiments.

**Fig 6:**
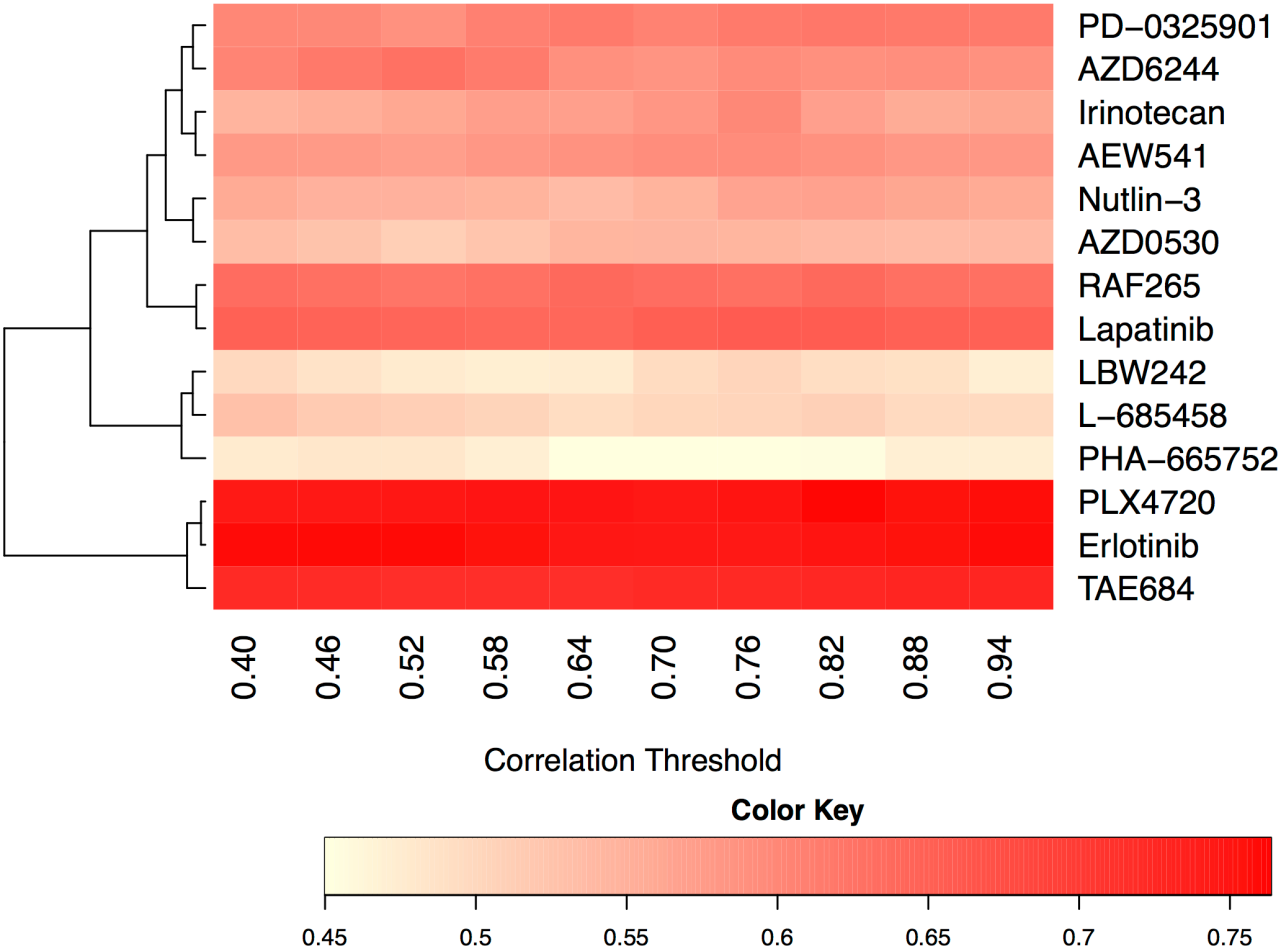
Heatmap using CCLE dataset for Balanced Accuracy of NBC using SVR. X-axis represents 10 different correlation thresholds, and y-axis represents 14 different drugs. Color intensity of the figure represents the BAC of each threshold and each drug combination.

Fig 6 presents the BAC heatmap of the results using CCLE dataset. To present the results in a readable form, we use hierarchical clustering [10] to organize the rows (i.e., drugs). Hierarchical clustering creates a tree structure of the hierarchy among drugs based on the similarity of the BAC values. This tree structure successively merges pairs of clusters (each cluster is a subset of drugs) until all clusters are merged into a single cluster. We perform clustering using the Euclidean distance between the vectors corresponding to the rows of this heatmap.

Our results suggest that gene expression profile based prediction is suitable for certain drugs but not for all. BACs of Erlotinib, PLX4720, and TAE684 drugs are the best three among 14 drugs regardless of the correlation threshold value. The BAC values of L-685458, LBW242, and PHA-665752 drugs are the worst among all. Overall, we observe that the NBC method is robust to varying values of the correlation threshold for all drugs.

**Fig 7:**
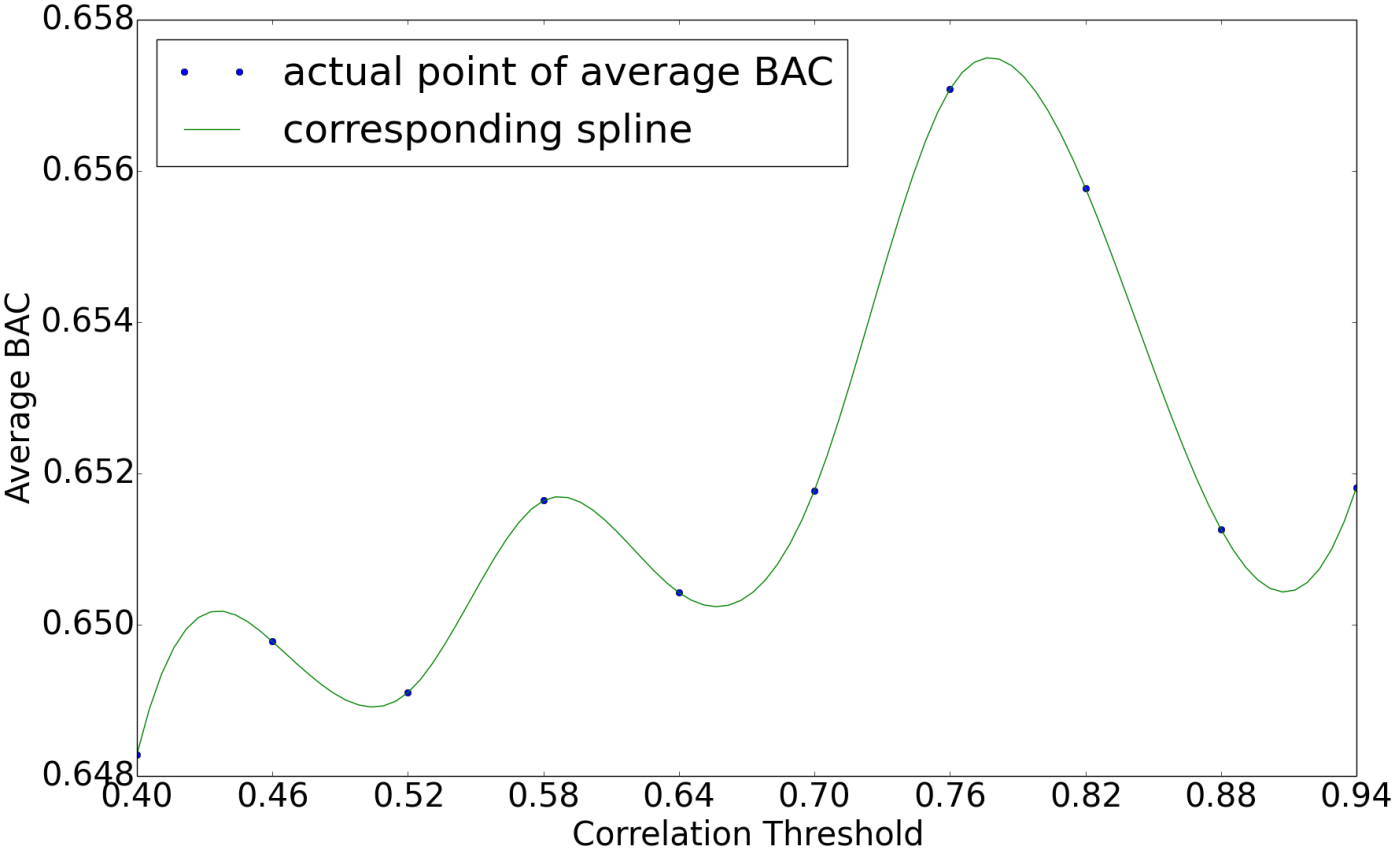
Line graph representing average BAC across drugs whose BAC is over 0.56 for each correlation threshold. X-axis represents 10 different correlation thresholds, and y-axis represents BAC value.

Next we focus on the drugs for which NBC yields highest average BAC values (those with BAC values over 0.56) to see how NBC’s accuracy varies with growing correlation threshold. To do that, we compute the average BAC value of all such drugs for each correlation threshold. Fig 7 plots the results. We observe that the BAC value first increases with growing correlation threshold value. After reaching to a peak value, it then tends to drop. The reason behind this is the following. When the correlation threshold is too small, the network model contains many false-positive edges. In other words even genes that have negligible correlation in transcriptional values contribute to the function that estimates a given gene’s transcription level. This leads to inaccurate estimations. On the other hand, when the correlation threshold is too stringent (i.e., too large), the predictor functions in the underlying network model loses key parameters for estimating the transcription level of genes. Such false dismissals can also result in reduced accuracy. Correlation threshold values around 0.75 to 0.8 yield the best results in our experiments.

### Evaluation of NBC for different pairs of drug and cancer type

In the previous section, we computed the accuracy of the NBC method for predicting the drug sensitivity over the entire CCLE dataset. This dataset contains samples belonging to various cancer types. The resistance of cells to a given drug can vary greatly depending tissue and cell type origin or the cancer. Thus, the accuracies reported in the previous section shows the average behavior of the NBC method across difference cancer types. An interesting question would be; how accurate are NBC and other predictors for individual cancer types? Here, we seek an answer to this question. Next, we describe how we do this.

**Fig 8:**
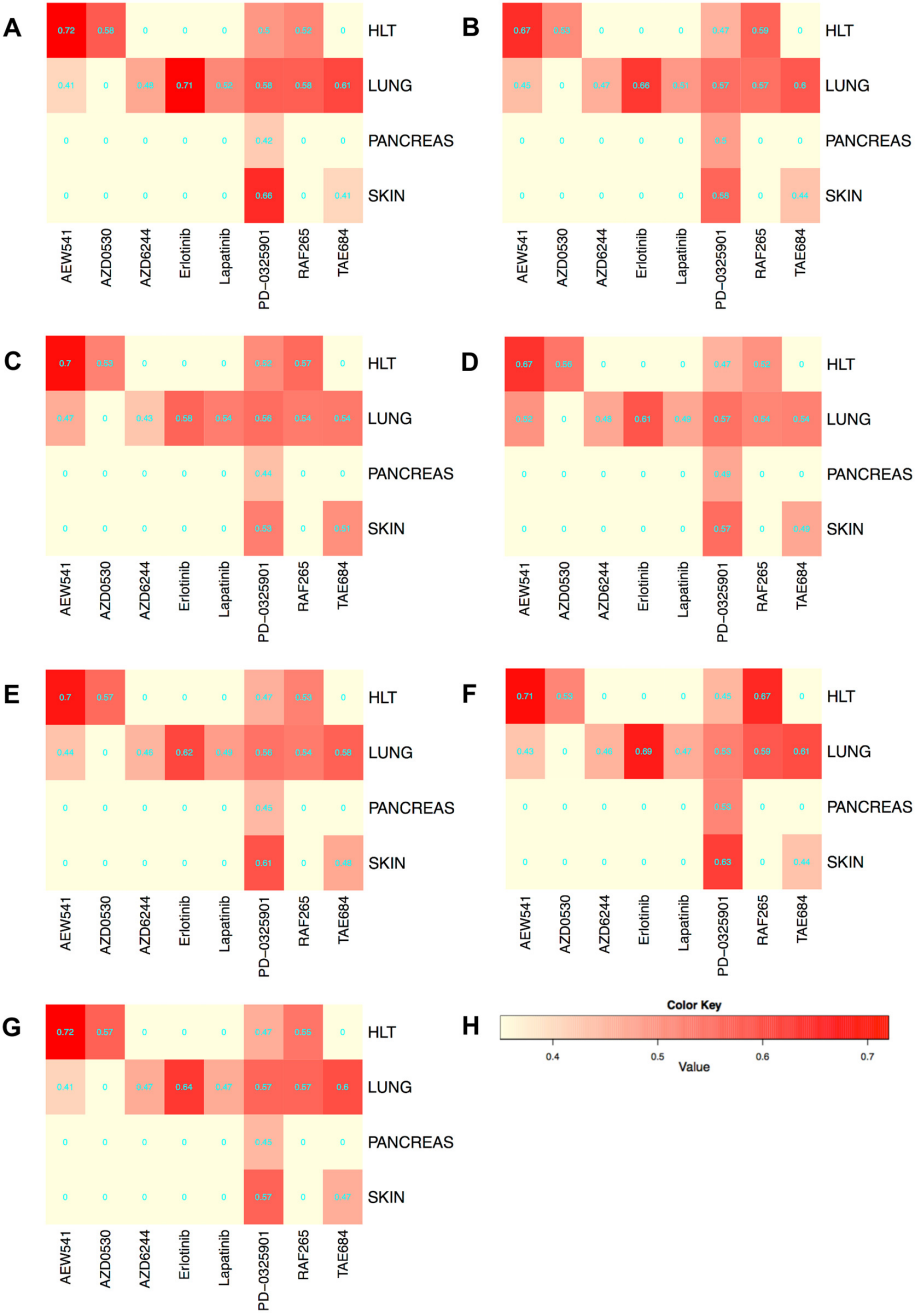
Heatmap using CCLE dataset for Balanced Accuracy. X-axis represents 8 different drug types, and y-axis represents 4 different cancer types. Color intensity of the figure represents the BAC of each drug and each cancer combination. (A)NBC(SVR). (B)NBC(Ridge). (C)SVM(linear). (D)SVM(RBF). (E)RF. (F)GNB. (G)kNN. (H)Color Key.

We only consider the drug/cancer type combinations for which we have sufficient number of samples for training and testing for each of the resistant and sensitive classes. We decide whether the number of samples is sufficient or not as follows. For each drug/cancer type combination, we perform double nested 5-fold cross-validation. We eliminate the combinations where the inner most validation split in nested CV contains either no resistant or no sensitive cell lines. After this filtering, only 8 drugs and 4 cancer types remain. The Table 2 shows the number of available cell lines for the combination of each drug and each cancer type in CCLE dataset. For each of the remaining combinations, we repeat the CV steps 100 times, each time with a different partitioning of the training and test samples and report the average balanced accuracy (BAC) of NBC. Fig 8 presents the results using CCLE dataset in heatmap form. In this figure, the rows and the columns denote cancer types and drugs respectively. Note that that some of the drug/cancer type combinations in this heatmap are excluded as well for they do not have sufficient number of samples. These are shown by the light color in the figure (e.g., Erlotinib and skin cancer combination).

**Table 2:** The number of cell lines used for the combination of each drug and each cancer type in CCLE dataset.

CancerType/Drug	AEW541	AZD0530	AZD6244	Erlotinib	Lapatinib	PD-0325901	RAF265	TAE684
HLT	37	28	0	0	0	31	41	0
LUNG	59	0	33	44	41	54	61	52
PANCREAS	0	0	0	0	0	25	0	0
SKIN	0	0	0	0	0	37	0	26

We observe that the accuracy varies across different drugs (i.e., columns) as well as cancer types (i.e., rows). For instance, we predict the resistance level to PD-0325901 in skin cancer better than other cancer types. Also, we are more accurate for predicting the resistance to Erlotionib than any other drug for the lung cancer. This suggests that, our method is more preferable for certain combinations of drugs and cancer types than others.

The results (Fig 8A) demonstrate that our method can yield higher prediction accuracy when it focuses on specific drug/cancer type combinations as opposed to training with the entire dataset. For example, BAC of the combination of AEW541 and HLT (0.72) is higher than that of AEW541 for overall cancer types (0.53—see Fig 1). However, this does not hold for all combinations. For instance, BAC of the combination of TAE684 and SKIN (0.39) is lower than that of TAE684 for overall cancer types (0.61—see Fig 1). We conjecture that such drop in prediction accuracy results from lack of sufficient number of training samples when we limit the training set to only those combinations. Thus, we expect that as more cell line data becomes available, the accuracy of those combinations would also increase drastically.

We also observe that the BACs of combination of AEW541/HLT, Erlotinib/Lung, and PD-0325901/Skin are the overall three highest for all predictors in Fig 8. NBC(SVR) is the best for predicting sensitivity of those combinations which has the BACs of 0.72, 0.71 and 0.66 respectively.

### Evaluation of the impact of feature selection strategies

Drug sensitivity prediction accuracy can vary for different sets of genes (i.e., features) used for the learning algorithm. Here, we explore this variation for several key feature selection strategies. One standard way to select these genes is to use the *χ*^2^ feature selection method. Note that this method purely uses the transcriptional variation between the two classes; resistant and sensitive (see Materials and Methods section). An alternative to this method is to exploit biological domain knowledge about the genes. This can be done in various ways. As the underlying challenge in this paper is to predict the resistance of cells to drugs that regulate cell death, we use the set of apoptotic genes (i.e., genes that belong to the Apoptosis network). We classify apoptotic genes as three subtypes which are pro-apoptotic, anti-apoptotic, and the union of these two sets. We compare statistical gene selection (*χ*^2^ method) with gene selections by biological domain knowledge. Random feature selection method takes a role of null hypothesis.

For each gene selection method and for each drug, we perform double nested 5-fold cross-validation and compute the average balanced accuracy (BAC). We repeat this 100 times each time with a different partitioning of the training and test samples and report the average result.

We have total 271 pro-apoptotic genes, 439 anti-apoptotic genes, and 661 combined-apoptotic genes. The reason why the number of combined-apoptotic genes is not equal to the sum of the number of pro and anti apoptotic genes is that some genes are considered as both pro and anti apoptotic. For all different gene selection methods, we pick *m*′ = 100 genes. We do this by picking the top 100 genes with the highest *χ*^2^ score.

**Fig 9:**
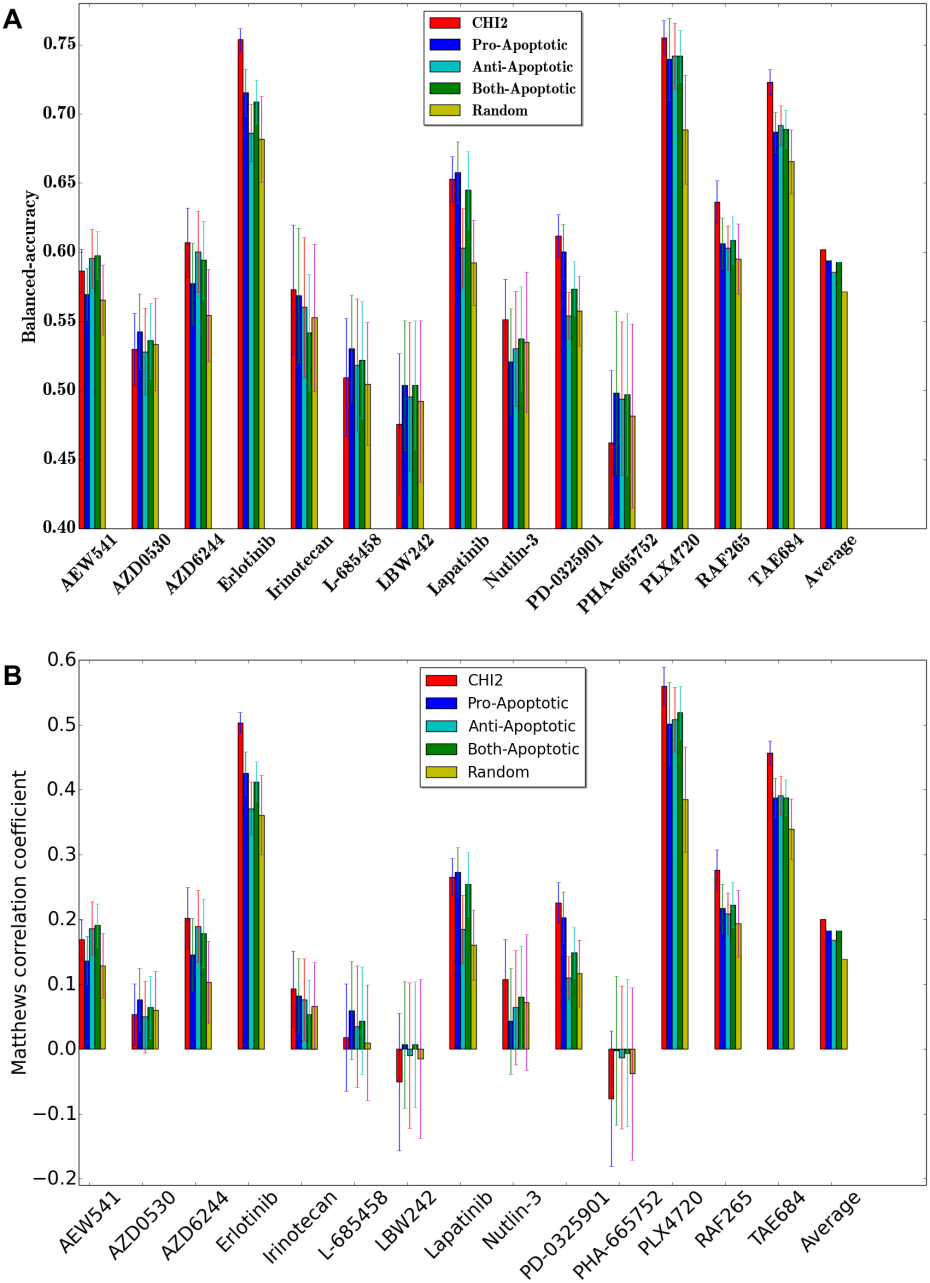
Performance comparison using CCLE dataset between *χ*^2^, pro-apoptotic, anti-apoptotic, both-apoptotic and random feature selections. X-axis represents drug names, and y-axis represents BAC. (A)Balanced Accuracy (BAC). (B)Matthews correlation coefficient (MCC).

Fig 9 presents the result using CCLE dataset. The result demonstrates that domain knowledge helps to select genes that improve prediction accuracy for a certain drug type. In 9 out of the 14 drugs, *χ*^2^ feature selection method is the winner among the five different feature selection methods. Pro-apoptotic feature selection yields the best accuracy in three different drugs, and anti-apoptotic feature selection is the best in two different drugs.

### Biological relevance of the drug sensitivity network

So far, we have demonstrated that we can determine whether a given transcriptome sample from cancer cell lines is resistant or sensitive to a large collection of well studied drugs. In this experiment we evaluate if our predictions are supported by the evidences existing in the literature. Below, we first explain how we conduct this experiment in detail. We then present the detailed results.

For each gene *g* and drug *d* combination, we calculate two values. The first one captures the number of publication evidences potentially indicating the use of drug *d* to target gene *g*. To compute this value, we search the NCBI (National Center for Biotechnology Information) PubMed database for the co-occurrence of the two keywords that is the combination of drug name and gene name. We call the number of publications that contain both keywords as the *publication evidence*. We conjecture that a large number of publications found this way implies that either the gene belongs to a regulatory pathway that is targeted by the drug or strongly affects the efficacy of the drug.

The second value we compute, named the *prediction success rate*, reflects the contribution of gene *g* in successful classification of test samples to resistant and sensitive classes for drug *d*. Conceptually, this can be viewed as the importance of *g* induced by the NBC method on *d*. More specifically, we compute this value as follows: Note that NBC constructs two network models for *d*, one for sensitive and one for resistant cell lines. Also, note that NBC predicts gene expression levels for all the genes using each of these two network models (see Materials and Methods section). For each gene *g*, we calculate the error introduced by the sensitive network model as the square of the difference between its predicted expression level of *g* and the actual expression level of *g* for each test sample. Let us denote the resulting error value with *ϵ*_*s*_. Similarly, we calculate the error introduced by the resistant network model and denote it with *ϵ*_*r*_. We say that gene *g* *contributes* to the correct classification of a test sample if the error of the true class of *g* is less than that of the other class. For instance, if a given test sample is resistant, *g* contributes only if *ϵ*_*r*_ < *ϵ*_*s*_. We compute the prediction success rate as the fraction of samples for which gene *g* contributes.

**Fig 10:**
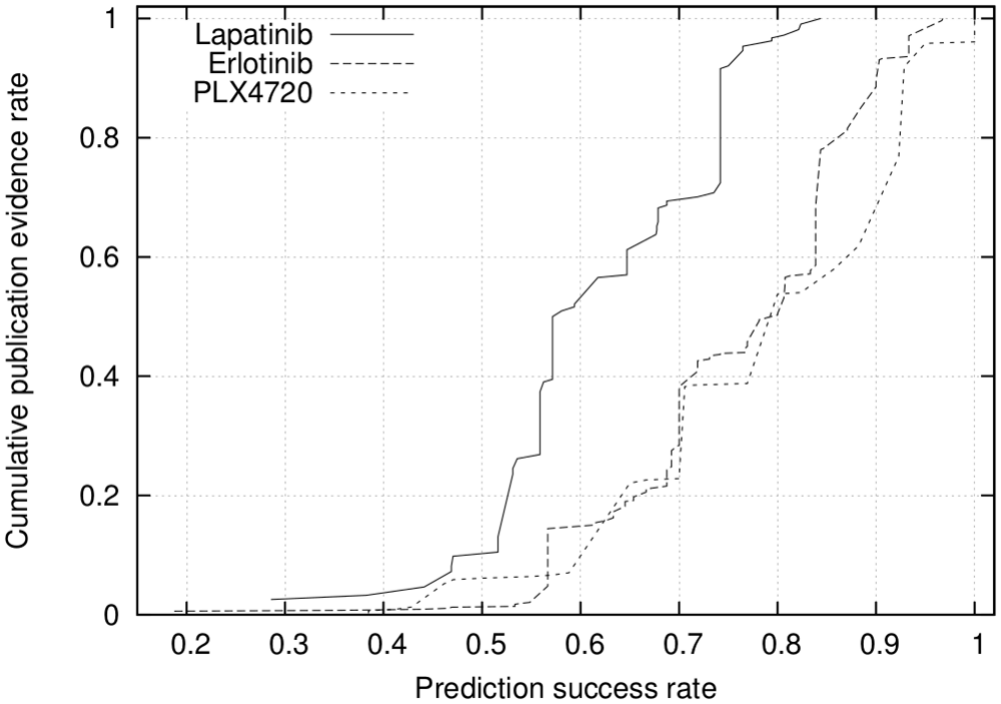
The relationship between prediction success rate of genes and their publication evidences for the top three drugs with the highest classification accuracy rate.

Ideally, we conjecture that genes that contribute more to the decision whether a cell line is resistant or sensitive to a given drug should have a large number of publication evidences. In order to test this conjecture, for each drug, we sort the genes in increasing order of their prediction success rates and compute the cumulative publication evidences. Note that, different drugs may appear in the literature with varying frequencies. To eliminate any bias introduced by such gap, we normalize the cumulative publication evidences of each drug to the [0, 1] interval by dividing it with the total publication evidence across all genes. Fig 10 presents the results for three drugs, Lapatinib, Erlotinib, and PLX4720. These are the three drugs for which the NBC method yields the highest accuracy.

Fig 10 demonstrates that the prediction success rate correlates with the publication evidence. The genes with low prediction success rates tend to have smaller increase in the publication evidences, while those with high prediction success rate tends to have steeper increase. For instance, for PLX4720 and Erlotinib, half of the publication evidences are for the genes with very high prediction success rates (i.e., ≥ 0.8 prediction success). This suggests that, high prediction success has great potential to guide future research on lesser studied drugs by limiting the studies to only those genes with highest prediction success rates.

## Discussion

In this paper we discussed a network based classifier method for predicting sensitivity of cell lines to anticancer drugs from transcriptome data. In the literature, this strategy has been used for predicting cancer types. Here, we extended it to estimate sensitivity of cells from different tumor types to various anticancer drugs. Instead of simply dividing cell lines equally into sensitive vs. resistant, we used clinical dose information to impart clinical relevance to the prediction. Our experimental results suggest that our NBC method outperforms existing classifiers in estimating sensitivity of cell lines for different drugs. If a prediction algorithm is to be used for predicting the sensitivity of particular cancer to chemotherapeutic drugs, it has to be able to select gene features. To this end, we explored and tested the application of domain specific knowledge by selecting genes that is known to regulate apoptosis. We also show that network models created by NBC method is biologically relevant and may be used to identify genes that are worth to be further investigated for certain anticancer drugs.

## Materials and Methods

In this section, we describe the dataset that we use in our experiments. The dataset contains the Cancer Cell Line Encyclopedia (CCLE) and the Genomics of Drug Sensitivity in Cancer (GDSC). We also describe how we use the NBC method for predicting drug sensitivity in tumor cell lines in detail. We then explain how we extend it to consider nonlinear associations among the transcripiton levels of genes. Next, we discuss how we integrate the domain knowledge to NBC in the form of the apoptotic genes. Finally, we summarize existing major classifiers and regression methods in the literature.

### Dataset

We use the Cancer Cell Line Encyclopedia (CCLE) dataset in our experiments (www.broadinstitute.org/ccle/home). The pharmacological profiles for 24 anticancer drugs across 479 of the cell lines available in this database was used in our study for prediction of cancer cell drug sensitivity. However, the number of cell lines available for each drug is different. The Table 3 and the Table 4 show the number of available cell lines for each drug in CCLE and GDSC dataset respectively. The Table 2 shows the number of available cell lines for the combination of each drug and each cancer type in CCLE dataset. These data sets were described in detail in Jordi Barretina et al [11]. For evaluation of performance of our method in addition to the CCLE dataset, we have also used Genomics of Drug Sensitivity in Cancer (GDSC) dataset which contains drug response data of about 700 cancer cell lines to 138 anticancer drugs [12].

**Table 3:** The number of cell lines used for each drug in CCLE dataset.

Drug	Number of Cell lines
AEW541	319
AZD0530	231
AZD6244	211
Erlotinib	203
Irinotecan	264
L-685458	106
LBW242	72
Lapatinib	246
Nutlin-3	77
PD-0325901	328
PHA-665752	56
PLX4720	105
RAF265	304
TAE684	264

**Table 4:** The number of cell lines used for each drug in GDSC dataset.

Drug	Number of Cell lines
AZD0530	565
PD-0325901	548
PHA-665752	232
PLX4720	483

To make our model relevant to clinical setting, drug concentrations, defined as the average *C*_max_ (uM) in blood plasma of patients taking the standard prescribed doses, for 15 drugs were obtained from literature search as shown in the Table 5. We took an approach to identify the average blood concentration of patients or healthy volunteers that were administered with the respective drug. By using the clinical concentration as the threshold, we make our predictions clinically relevant. Log transformed values of the clinical concentrations −log(*C*_max_ × 1.5) + 6 and −log(*C*_max_/1.5) + 6 were used as resistant and sensitive cutoffs for the experiments respectively. These two clinical cutoff values are to determine the sensitivity class of a cell line to a given drug, one for drug resistant and the other for sensitive class. Table 5 lists the drugs available in the Cancer Cell Line Encyclopedia (CCLE) dataset, their two clinical cutoff values, and their literature where these cutoff values were obtained from. We do not have reliable clinical concentrations for the drug whose literature is ‘From statistical data’. For those drugs, we instead extract clinical cutoff values from statistical data as follows. EC50 is defined as the effective concentration at which the drug exhibits its half-maximal activity. EC50 value of each cell line is recorded in CCLE and GDSC dataset. Let us denote the mean and the standard deviation of the EC50 value of such a drug across all cell lines with *μ* and *σ* respectively. We compute the resistant and sensitive cutoffs as −log(*μ* ± 0.3 * *σ*).

**Table 5:** List of resistant and sensitive cutoffs from the drug concentration values.

Drug	Resistant	Sensitive	Supporting literature
17-AAG	5.000	5.002	[13]
AEW541	5.299	5.615	From statistical data
AZD0530	5.910	6.262	[14]
AZD6244	5.618	5.970	[15]
Erlotinib	5.562	5.915	[16]
Irinotecan	5.592	5.944	[17]
L-685458	5.290	5.626	From statistical data
LBW242	5.605	6.003	From statistical data
Lapatinib	5.579	5.931	[18]
Nilotinib	5.593	5.945	[19]
Nutlin-3	5.336	5.512	From statistical data
PD-0325901	6.269	6.621	[20]
PD-0332991	6.186	6.539	[21]
PF2341066	6.045	6.397	[22]
PHA-665752	5.303	5.616	From statistical data
PLX4720	5.392	5.682	From statistical data
Paclitaxel	5.000	5.267	[23]
Panobinostat	6.541	6.894	[24]
RAF265	5.718	6.125	From statistical data
Sorafenib	5.000	5.110	[25]
TAE684	5.477	5.755	From statistical data
TKI258	5.925	6.277	[26]
Topotecan	7.563	7.916	[27]
ZD-6474	6.219	6.571	From statistical data

Let us denote the EC50 of a given cell line for a drug with *E*. If the −log(*E*) is above the sensitive cutoff, we call this cell line to be *sensitive* to that drug. Likewise, if the −log(*E*) is below the resistant cutoff, we say that this cell line is *resistant* to that drug. If the −log(*E*) is between resistant and sensitive cutoffs, that cell line does not clearly belong to any of the two classes. We ignore such cell lines in our experiments.

Among the 24 drugs in the Cancer Cell Line Encyclopedia (CCLE) dataset, the distribution of resistant and sensitive cell lines of 10 drugs (17-AAG, Nilotinib, PD-0332991, PF2341066, Paclitaxel, Panobinostat, Sorafenib, TKI258, Topotecan, ZD-6474) is significantly biased to either almost resistant or almost sensitive classes. As a result, one of the two classes have too few samples to train any learning model. We do not consider these 10 drugs in our experiments to ensure reliable results. For Genomics of Drug Sensitivity in Cancer (GDSC) dataset, we first consider drugs that exist in both CCLE and GDSC. Then, we exclude drugs for which the distribution of resistant and sensitive cell lines is significantly biased to either almost resistant or almost sensitive classes. Finally, we consider only AZD6244, PD-0325901, PHA-665752, and PLX4720 in GDSC dataset.

### Network-based Classifier (NBC)

Expression levels of genes provide key clues about how the cells function. This is because not only they impact almost all major biological processes within cell but also they are influenced by extra cellular signals. As a result, many machine learning techniques use gene expression levels as features for predicting different characteristics of cells. Network Based Classifier (NBC) [28], Support Vector Machines (SVM) [29], and Random Forest (RF) [30] are just a few examples to these methods.

Unlike most of the classical machine learning approaches, NBC exploits the interaction among genes through their expression levels. As a result, it often yields more accurate prediction than most of the other existing methods in the literature. In this paper, we adapt the NBC algorithm to predict the drug sensitivity level of a given cell line based on the expression levels of its genes. More specifically, for each cell line and drug combination, we classify that cell line into one of the two classes: *resistant* or *sensitive* depending on the EC50 value of that drug for that cell line. If the minus-log EC50 value is above a user defined sensitivity cutoff, we say that it is sensitive. If that value is below a user defined cutoff, we say that it is resistant. If it is between those two cutoff values than we cannot clearly say whether it is sensitive or resistant. We elaborate on how these cutoffs are determined in the section named ‘Dataset’ in Results.

In the following, we first take a small detour to describe how the NBC method works. We then explain how we extend it to handle nonlinear associations among the genes to tackle the problem considered in this paper.

#### The NBC algorithm

This algorithm has three major steps; (1) selection of features, (2) building a network model for each class, (3) learning functions to predict transcription levels of genes. We summarize these steps next.

**Step 1: Feature selection** Similar to most transcription datasets, the transcriptome from the CCLE database contains a large number of genes (over 18,000). Using all of these genes to predict a cell characteristic, such as drug sensitivity, is not feasible for several reasons. First, the number of genes (i.e., features) is much larger than the number of samples (e.g., cell lines). Learning in such a high dimensional space possibly will lead to overfitting, and yield inaccurate predictions. Second, most of these genes have no influence on the sensitivity of cells to drugs. Thus, it is desirable to select only a small subset of features that are relevant and have high predictive power.

NBC uses the *χ*^2^ method [9] to select features as follows. Assume that we are given a set of *n* samples, where each sample contains the expression levels of *m* genes. For each gene *g*_*i*_, we construct a matrix *A*^*i*^ with two rows. The first and the second rows of this matrix correspond to the samples in the resistant and sensitive classes respectively. Next, we describe how we construct the first row this matrix for the resistant samples. The second row for the sensitive samples follow the same pattern. We quantize the expression level of *g*_*i*_ in all resistant samples by partitioning the range of gene expression values observed in the dataset for *g*_*i*_ into fixed length intervals. We call each of these intervals a *term*. Let us denote the number of terms with *t*. We say that a sample is assigned to a term if its gene *g*_*i*_ has an expression value in the corresponding interval. For each term, we count the number of resistant samples with gene *g*_*i*_ assigned to that term. We set the value of each entry in the first row of *A*^*i*^ to the count corresponding to that term. Thus, for gene *g*_*i*_, for each class *c* ∈ {1, 2} (i.e., resistant and sensitive respectively), and each term *j*, the entry $A_{c,j}^{i}$ shows the number of samples in class *c* which are assigned to the *j*th term.

We maintain the expected frequency of all class and term combinations for gene *g*_*i*_ in matrix *E*^*i*^ which has the same size as *A*^*i*^. Next, we discuss how we compute this matrix. We compute the probability of observing the *j*th term for gene *g*_*i*_ as the fraction of samples which are assigned to that term (i.e., ($A_{1,j}^{i}$+$A_{2,j}^{i}$)/*n*). We denote this with $P_{\text{term}}^{i}\left(j\right)$. Similarly, we compute the probability of observing the *c*th class for gene *g*_*i*_ as the fraction of samples belonging to that class (i.e., ($\sum_{j=1}^{t}A_{c,j}^{i}/n$). We denote this with $P_{\text{class}}^{i}\left(c\right)$. We next compute the expected frequency of $A_{c,j}^{i}$ as $E_{c,j}^{i}=n\times P_{\text{term}}^{i}\left(j\right)\times P_{\text{class}}^{i}\left(c\right)$. Finally, we compute the *χ*^2^ value for gene *g*_*i*_ as $\sum_{j=1}^{t}\sum_{c=1}^{2}\frac{{\left(A_{c,j}^{i}-E_{c,j}^{i}\right)}^{2}}{E_{c,j}^{i}}$.

Notice that, *χ*^2^ method tests the independence between two events; in our problem the independence between the term and the class of samples. The higher the *χ*^2^ value is, the more dependent the occurrence of the given term and class pair are. Thus, one can be used to predict the other. Once we compute the *χ*^2^ value for all genes, we select the top 100 genes with the highest *χ*^2^ values.

**Step 2: Constructing network models** At the end of Step 1, we have a small number of genes (i.e., we choose top 100 genes in our experiments) whose expressions can explain the sensitivity levels of cells to drugs the best. Let us denote the number of genes selected with *m*′. In this step, we construct a network model for each of the two classes, sensitive and resistant. Briefly, each model describes the relationship between the transcription levels of genes for the class corresponding to that model. More specifically, let us denote the set of genes selected in Step 1 with G= {*g*_1_, *g*_2_, …, *g*_*m*′_}. Assume that the number of cell lines in the sensitive and resistant class for a given drug is *s* and *r* respectively. Let us denote the set of cell lines in the sensitive class with {*c*_1_, *c*_2_, …, *c*_*s*_} and those in the resistant class with {$c_{1}^{\prime }$, $c_{2}^{\prime }$, …, $c_{r}^{\prime }$}. Each gene $g_{i}\in G$ defines two vectors, one for sensitive and one for resistant class, denoted with *e*_*i*_ and $e_{i}^{\prime }$. The *j*th entry in *e*_*i*_ is the transcription level of *g*_*i*_ in cell line *c*_*j*_. Similarly, the *j*th entry in $e_{i}^{\prime }$ is the transcription level of *g*_*i*_ in cell line $c_{j}^{\prime }$.

Now that we have defined the two vector sets, we are ready to construct the network models for sensitive and resistant classes. In the following, we first describe how we construct the graph model for the sensitive class. We denote this graph with *G*_*S*_ = (*V*,*E*_*S*_). Here ∀*i*, 1 ≤ *i* ≤ *m*′, node *v*_*i*_ ∈ *V* corresponds to gene *g*_*i*_. For all pairs of genes *g*_*i*_, $g_{j}\in G$, we compute the Pearson’s correlation coefficient between the pair of vectors *e*_*i*_ and *e*_*j*_. If the absolute value of this correlation is above a user specified threshold *ϵ* then we say that *g*_*i*_ and *g*_*j*_ are *correlated* in the sensitive class. We draw an undirected edge between *v*_*i*_ and *v*_*j*_ and insert it to *E*_*S*_ if *g*_*i*_ and *g*_*j*_ are correlated in the sensitive class.

We construct the graph model for the resistant class similarly. We denote this graph with *G*_*R*_ = (*V*,*E*_*R*_). The only difference between this graph and *G*_*S*_ is that for all gene pairs *g*_*i*_, $g_{j}\in G$, we use the vectors $e_{i}^{\prime }$ and $e_{j}^{\prime }$ instead of *e*_*i*_ and *e*_*j*_ to compute their correlation. Similarly, if the absolute value of this correlation is above a user specified threshold *ϵ* then we say that *g*_*i*_ and *g*_*j*_ are correlated in the resistant class. We draw an undirected edge between *v*_*i*_ and *v*_*j*_ and insert it to *E*_*R*_ if *g*_*i*_ and *g*_*j*_ are correlated in the resistant class.

**Step 3: Learning predictor functions** At the end of Step 2, we have two graph models, *G*_*S*_ and *G*_*R*_ for sensitive and resistant classes respectively. We construct two predictor functions for each *v*_*i*_ ∈ *V*, one for *G*_*S*_ and the other for *G*_*R*_ using the edges incident to *v*_*i*_ in *E*_*S*_ and *E*_*R*_ respectively. These functions follow the conjecture that correlated genes can explain the transcription levels of each other. More specifically, NBC uses Ridge regression for this purpose. For each node *v*_*k*_ ∈ *V*, let us denote the matrix that consists of vector sets *e*_*i*_ as column vectors for all *i* ∈ {*i* |(*v*_*i*_, *v*_*k*_) ∈ *E*_*S*_} with *A*_*k*_ for the sensitive class. Similarly, let us denote the matrix that consists of vector sets $e_{i}^{\prime }$ as column vectors for all *i* ∈ {*i* |(*v*_*i*_, *v*_*k*_) ∈ *E*_*R*_} with $A_{k}^{\prime }$ for the resistant class.

We derive a regression model *F*_*k*_ that predicts *e*_*k*_ from matrix *A*_*k*_ for each node *v*_*k*_ in sensitive class, and another one $F_{k}^{\prime }$ that predicts $e_{k}^{\prime }$ from matrix $A_{k}^{\prime }$ for each node *v*_*k*_ in resistant class. NBC uses Ridge regression with regularization for this purpose. Regularization solves an ill-posed problem or prevents overfitting by introducing a form of a penalty for complexity. Ridge regression is a type of linear regression that uses regularization technique. It has a penalty parameter *λ*. We use *λ* = 10^−3^ for all predictor functions globally. We create Ridge regression model [31] that predicts *e*_*k*_ from matrix *A*_*k*_ for each node *k* in sensitive class, and similarly $e_{k}^{\prime }$ from matrix $A_{k}^{\prime }$ for each node *v*_*k*_ in resistant class.

#### Learning non-linear predictor functions

Though time complexity of NBC using Ridge regression is low, it is limited to the transcription of linear relationships among genes. Therefore, it is not expressive enough to describe the complex relationships that govern the process of genes’ regulation of each other. Here, we extend the NBC algorithm to capture non-linear associations among genes. We adopt Support Vector Regression (SVR) [32] for this purpose. SVR learns a non- linear regression model by using the kernel trick. The regression model is a linear function in the space induced by the kernel. However, that linear function corresponds to a non-linear function in the original space. We create a SVR model that predicts *e*_*k*_ from matrix *A*_*k*_ for each node *v*_*k*_ in sensitive class, and similarly $e_{k}^{\prime }$ from matrix $A_{k}^{\prime }$ for each node *v*_*k*_ in resistant class. Unlike the NBC method, we use the radial basis function (RBF) [33] as a kernel to transform the problem from non- linear to a linear space. This requires using three parameters. (i) *γ* is a kernel coefficient for radial basis function (RBF). (ii) *C* is a penalty parameter. This parameter controls the trade-off between the error obtained on the training data and margin maximization. (iii) *ϵ* specifies the epsilon-tube within which no penalty is associated in the loss function.

In NBC using SVR, each predictor function of a node *v*_*k*_ has flexible parameters to maximize the capability to capture variable non-linear characteristics. To search the best parameters of SVR for the predictor function of each node *v*_*k*_, we use a grid-search method. This method exhaustively searches through a manually specified subset of parameter space. We use the following parameter space: *C* ∈ {10^−1^, 10^0^, 10^1^}, *γ* ∈ {10^−1^, 10^0^, 10^1^}, and *ϵ* ∈ {10^−1^}. We use 5-fold cross-validation to find the best parameters of the predictor function of a node *v*_*k*_.

For each node *v*_*k*_ ∈ *V*, *F*_*k*_ with different parameters will give a different predicted gene expression level. We consider the parameters that yield the predicted gene expression level closest to the actual gene expression level as the best. In this way, we compute the parameters of each *F*_*k*_, and those of $F_{k}^{\prime }$ for sensitive and resistant network models respectively. Thus, this strategy requires three nested loops for each predictor function *F*_*k*_ and $F_{k}^{\prime }$ to tune the best parameter in SVR while no loop is required to tune the best parameter in Ridge regression. Nevertheless, we conjecture that NBC using SVR has great potential to capture non-linear associations among genes and thereby to outperform NBC using Ridge regression for predicting drug sensitivity.

#### Predicting drug sensitivity using NBC

Once the NBC method constructs the network models and the corresponding predictor functions, we are ready to use to to classify new samples into resistant or sensitive classes. Briefly, we do this as follows. NBC considers a new sample as a vector that consists of gene expression levels of the *m*′ genes selected by the *χ*^2^ method described above. Let us denote this vector with *x* = [*x*_1_, *x*_2_, …, *x*_*m*′_]. For all *k* (1 ≤ *k* ≤ *m*′) NBC makes two predictions; one assumes that the test sample is sensitive and the other assumes that it is resistant. The function *F*_*k*_ predicts the expression level of the *k*th gene in this vector as *y*_*k*_ = *F*_*k*_(*x*) should the test sample be in the sensitive class. Similarly, the function $F_{k}^{\prime }$ predicts the expression level of the *k*th gene as $y_{k}^{\prime }=F_{k}^{\prime }\left(x\right)$ if it was in the resistant class. Of these two predictions, the more accurate one will be the one corresponding to the true class of the test sample. Thus we construct two vectors *y* = [*y*_1_, *y*_2_, …, *y*_*m*′_] and $y^{\prime }=\left[y_{1}^{\prime },y_{2}^{\prime },\ldots ,y_{m^{\prime }}^{\prime }\right]$ for sensitive and resistant classes. We then calculate the mean square error (MSE) for the sensitive and network model as $\frac{1}{m^{\prime }}\;\sum_{k=1}^{m^{\prime }}{\left(x_{k}-y_{k}\right)}^{2}$, and that for the resistant network model as $\frac{1}{m^{\prime }}\;\sum_{k=1}^{m^{\prime }}{\left(x_{k}-y_{k}^{\prime }\right)}^{2}$. We predict the class of the test sample as the one with less mean square error (MSE).

### Apoptotic genes

Apoptosis is a genetically controlled mechanism that leads to the suicide of the cell. The cellular basis of many anticancer drugs are to induce apoptosis of cancer cells. A number of genes that encode anti or pro apoptotic regulators, have known to function as oncogenes or tumor suppressor genes, respectively [34]. Expression status of pro-apoptotic and anti-apoptotic genes could potentially predict sensitivity to anticancer drugs. Human genes annotated with Gene Ontology IDs 0043065 and 0043066 were extracted as pro-apoptotic and anti-apoptotic gene lists respectively and filtered by ‘Direct Annotation’. We use the list of apoptotic genes for a feature selection exploiting biological domain knowledge. Then, we compare the prediction performance between a statistical feature selection method such as *χ*^2^ and a feature selection using apoptotic genes in the section named ‘Evaluation of the impact of feature selection strategies’ in Results.

### Overview of the existing classifiers

In this section, we summarize four state of the art classifiers in the literature, namely SVM [29], Random Forest (RF) [30], Naive Bayes [35], and K-nearest Neighbor (kNN) classifier [36]. We compare our method with these four methods in our experiments.

#### Support Vector Machines (SVM)

The SVM machine learning algorithm has been used frequently in many fields such as bioinformatics, text recognition, image recognition. It is inherently a binary, and discriminative classifier. Two key components that define SVM are a maximum margin hyperplane and kernel trick [29]. SVM finds a maximum margin hyperplane that separates the given training data points into two half-spaces lying on each side of the hyperplane. Support vectors are the data points which reside closest to the maximum margin hyperplane. Typically, Lagrange multipliers are used to find the maximum margin hyperplane analytically.

Linear SVM assumes that the data points are linearly separable. When this assumption fails, we exploit kernel trick to find a non-linear hyperplane. The basic idea of finding non-linear hyperplane is to map the original feature space to higher dimensional feature space in which the data points are linearly separable. Common kernel functions are polynomial, radial basis function (RBF), and sigmoid.

#### Random Forest (RF)

Random Forest (RF) is an ensemble method that uses multiple decision trees [30]. The decision tree consists of internal and leaf nodes. Each internal node has a splitting rule and each leaf node is labeled with a class. The decision tree algorithm uses information gain as a splitting criterion. Information gain shows how important a given feature is. In other words, most useful features for discriminating between the classes to be learned are determined by information gain. Thus, the feature with the highest information gain is embedded at the root node. Branching occurs at each internal node after making its decision until it reaches a leaf node.

The disadvantage of decision tree algorithm is that small variations in the data result in a totally different tree being generated. RF overcomes this disadvantage by exploiting bootstraping. More specifically, RF constructs multiple decision trees by bootstraping samples. To predict the class of a test sample, the algorithm uses all these multiple trees in the way that takes the majority vote over all the decision trees.

#### Naive Bayes (NB)

Given a class variable *y* and a feature vector {*x*_1_, …, *x*_*n*_}, Bayes’ theorem states the following relationship: $P\left(y|x_{1},\ldots ,x_{n}\right)=\frac{P\left(y\right)P\left(x_{1},\ldots ,x_{n}|y\right)}{P(x_{1},\ldots ,x_{n})}$

Briefly, NB is based on Bayes’ theorem with the assumption that independence exists among all features. It is difficult to learn the joint probability *P*(*x*_1_, …, *x*_*n*_|*y*) in Bayes method without this assumption. With this assumption, we write *P*(*y*|*x*_1_, …, *x*_*n*_) as $\frac{P\left(y\right)\prod_{i=1}^{n}P(x_{i}|y)}{P\left(x_{1},\ldots ,x_{n}\right)}$.

Since *P*(*x*_1_, …, *x*_*n*_) is constant given the data, NB uses the following classification rule:

$$\hat{y}=\underset{y}{\mathrm{argmax}}\left(\;P\left(y\right)\prod_{i=1}^{n}P\left(x_{i}|y\right)\;\right)$$

There are different NB classifiers depending on which distribution of *P*(*x*_*i*_|*y*) we use. In our study, we use Gaussian distribution as it is one of the most commonly used distribution.

#### K-Nearest-Neighbor (kNN)

kNN is an instance-based learning algorithm which compares new test samples with those samples whose classes are already known [36]. More specifically, it computes the distance between the given test sample and the remaining samples. It then classifies the test sample into the class where the majority of its *K* nearest neighbors agrees. In general, the distance function depends on the underlying application. That said, after it uses the euclidean distance for this purpose, we also use this distance function in our experiments. Unlike other learning algorithms, kNN induces its hypothesis directly from training instances. As a result, hypothesis complexity increases with the size of the training data.

### Overview of the existing regression methods

#### Bayesian multitask multiple kernel learning (BMTMKL)

Bayesian multitask multiple kernel learning (BMTMKL) method [3] exploits four machine learning principles: kernelized regression, multiview learning, multitask learning, and Bayesian inference. Kernelized regression can capture non-linear relationships between genomic features, and drug sensitivities of cell line. Multiview learning principle integrates heterogeneous input data into a single model. Multitask learning is the sharing of information between drugs, which implies simultaneous modeling of drug sensitivities across all the drugs. Additionally, Bayesian inference learned all model parameters to handle the uncertainty from the small sample size.

#### Elastic Net (EN)

Elastic Net regression is a hybrid approach that blends both penalization of the L2 and L1 norms. The combination of both penalization of the L1 and L2 allows that the model is sparse where few of the weights are non-zero, and the model also maintains the regularization properties of Ridge regression. Elastic Net is particularly useful when the number of predictors is much bigger than the number of observations [37], and when there are multiple predictors which are correlated with one another.
